# Under the Covers: The Effect of a Temperature-Controlled Mattress Cover on Sleep and Perceptual Measures in Healthy Adults

**DOI:** 10.3390/clockssleep7040055

**Published:** 2025-10-01

**Authors:** Shauna Stevenson, Haresh Suppiah, Toby Mündel, Matthew Driller

**Affiliations:** 1 Sport, Performance, and Nutrition Research Group, School of Allied Health, Human Services and Sport, La Trobe University, Melbourne 3086, Australia; h.suppiah@latrobe.edu.au; 2 Department of Kinesiology, Brock University, St. Catharines, ON L2S 3A1, Canada; tmundel@brocku.ca

**Keywords:** temperature manipulation, sleep quality, sleep technology, thermal comfort, sleep environment, bedding, mattress

## Abstract

Ambient temperature and thermoregulation influence sleep quality. This study investigated the effects of a temperature-controlled mattress cover on sleep and perceptual outcomes in healthy adults. In a randomised, counterbalanced, crossover design, 34 healthy adults (20 F, 14 M; age, 30 ± 5 y) used a temperature-controlled mattress cover for 14 nights, following ≥3 nights of familiarisation. The temperature feature was on for 7 nights (POD) and off for 7 nights (CON). Sleep was assessed via wrist actigraphy, while heart rate (HR), heart rate variability (HRV), and respiratory rate (RR) were recorded by embedded sensors in the mattress cover. Participants completed daily and weekly questionnaires evaluating sleep quality, thermal comfort, and thermal sensation. Linear mixed models showed significant main effects of condition favouring POD over CON for all daily perceived outcomes (all *p* < 0.05). A large, significant improvement in perceived sleep quality was observed (*p* = 0.001, d = 0.92). No significant differences were found in objective sleep metrics or biometric measures (all *p* ≥ 0.05). A temperature-controlled mattress cover was associated with improved subjective sleep quality and thermal-related perceptions despite minimal changes in objective or biometric outcomes, which may in part reflect expectancy, or placebo effects. Further research is needed to explore whether these perceptual benefits lead to physiological improvements over time.

## 1. Introduction

Previous work has reported that sleep is sensitive to even slight variations in nighttime ambient temperature [1], with laboratory studies demonstrating that thermal discomfort—whether in hot or cold conditions—reduces total sleep time, delays sleep onset latency, and increases minutes spent awake after sleep onset [2,3]. Additionally, challenging thermal environments and impaired thermoregulation are some of the primary causes of sleep disturbances [4]. Advances in technology now allow for real-time adjustments in bed temperature while simultaneously tracking sleep parameters and biometric data. Enabling individuals to regulate their thermal environment may offer a novel approach to improving sleep quality, but there has been limited research conducted in this area.

In the hours preceding sleep, the increasing homeostatic drive for sleep is accompanied by a decline in core body temperature ($T_{core}$) to ~36.6 °C, facilitating peripheral heat loss and an increase in distal skin temperature, supporting sleep initiation [4,5]. $T_{core}$ continues to decrease during sleep, reinforcing sleep duration [6]. Optimal sleep occurs when skin temperatures remain within 33.5 °C to 35.5 °C [7,8], with deviations disrupting continuity and reducing total sleep time [4,9,10]. Thermoregulatory strategies to promote sleep include passive body heating before bedtime (e.g., warm showers) [11], electric blankets [12], and temperature-controlled bedding systems. More recently, innovative bedding technologies have been developed to modulate thermoregulation in applied settings. High-heat-capacity mattresses, for instance, have been shown to increase deep sleep (N3 and slow-wave energy) and reduce resting heart rate in healthy young male participants [13], while also lowering $T_{core}$ and improving subjective sleep quality [14].

Another approach is a sleep system consisting of a selective thermal stimulation pillow and a dual-temperature zone mattress [15]. This technology applies both heating (via pillow and outer mattress edges) and cooling (central mattress zone) at the same time [15]. When applying mild heating to the cervical spine region of healthy young males for 30 min after the lights were turned off, it was reported that this condition significantly enhanced heat dissipation, decreased $T_{core}$, shortened sleep latency, and improved subjective sleep quality compared to a control condition [15]. However, technologies now allow users to tailor the temperature within their bed microclimate. Other interventions to target thermoregulation include the use of high-heat-capacity mattresses, which passively absorb the body heat of their users during the early part of the night. Such technology has led to significant reductions in resting heart rate and enhanced sleep quality while decreasing core body temperature [13,14]. This technology has also been utilised in athletes, but no significant improvements were observed with a high-heat-capacity mattress [16] or when comparing a low-heat-capacity mattress and a high-heat-capacity mattress [17]. However, further research is warranted in the general population, who have differing thermoregulatory needs.

Temperature-controlled mattress covers, or toppers, have been developed to provide an individualised thermal environment by allowing users to customise the temperature of the bed microclimate. Moyen et al. [18] investigated the use of a temperature-controlled mattress cover in 54 healthy adults over 16 days (7 nights with the temperature-regulation setting turned off and 7 nights with the temperature-regulation setting turned on, allowing users to customise their bed microenvironment, followed by another 2 nights with the setting turned off). Results showed that when the mattress cover was turned on, deep and light sleep increased by 14 and 23 min, respectively, and was also associated with an increased total sleep duration of 10 min. Further, sleeping heart rate (−2%) and heart rate variability (+ 7%) were significantly improved with the mattress cover technology [18]. To our knowledge, this study is the first to evaluate this technology, and while initial results are promising, independent verification is required. Further, research utilising this device in a randomised, counterbalanced crossover design, with familiarisation and controlled for external factors in the sleep environment (e.g., ambient temperature) in the general population, is required.

Therefore, the current study aimed to determine whether a temperature-controlled mattress cover may influence outcome measures of sleep metrics (total sleep time, wake after sleep onset, sleep onset latency, sleep efficiency, and sleep regularity), biometric measures (heart rate, heart rate variability, and respiratory rate), perceived outcomes on sleep (including calmness of sleep, ease of falling asleep and waking, being refreshed after waking, and sleep satisfaction), as well as thermal sensation and thermal comfort in healthy adults across 14 nights.

## 2. Results

Throughout the data collection period, the average room temperature and relative humidity of the participants’ sleep environments (during each individual’s sleep window across both conditions) were 21 ± 3 °C and 60 ± 7% rH, respectively, in both the control condition (CON) and the temperature regulation condition (POD). There were no significant differences in room temperature or relative humidity between conditions (*p ≥* 0.05). A total of 68% (*n* = 23) of participants reported changing the POD temperature via the device’s AI autopilot feature or the device’s dedicated phone application (when in POD condition).

Of the 34 participants, 25 (74%) reported making at least one manual adjustment to the bed temperature during the study via the dedicated phone application, while 9 (26%) either did not adjust or relied solely on the autopilot function. Most adjustments were minor (±1–3 levels; please see methods for description of levels) and were made in response to feeling too cold (e.g., on colder nights, early mornings, or following drops in ambient temperature). Fewer participants reported lowering the temperature due to feeling too warm. Several described increasing the bedtime phase or early-night phase setting before subsequently reducing it once in bed, whereas others made only occasional, single-night adjustments.

### 2.1. Menstrual Cycle Questionnaire

Based on responses to the menstrual cycle questionnaire, 19 out of the 20 female participants reported a regular cycle length of 25–35 days, with one participant reporting an irregular cycle. Two participants had a diagnosed reproductive or menstrual disorder. Four participants were using oral contraceptives or other hormonal contraceptive methods.

### 2.2. Daily Measures

**Figure 1:**
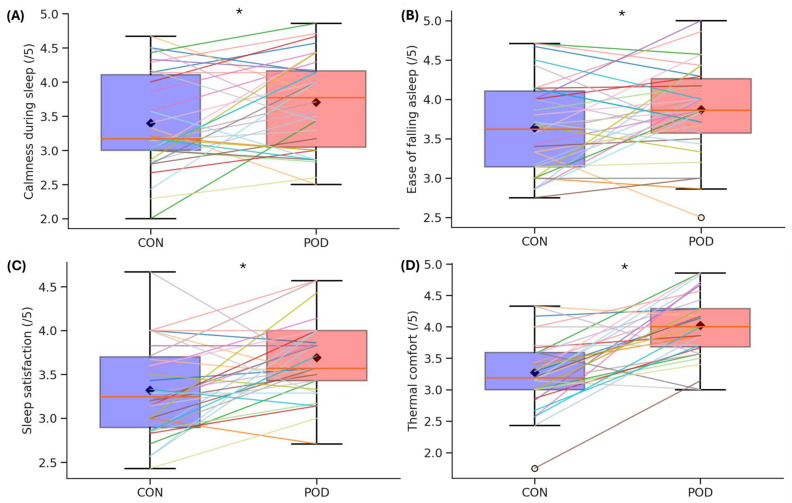
Box plots showing the differences in subjective daily perceived outcomes, including (**A**) calmness of sleep, (**B**) ease of falling asleep, (**C**) sleep satisfaction, and (**D**) thermal comfort, in the CON and POD conditions. Each box represents the interquartile range (IQR), with the horizontal line inside the box indicating the median with error bars extending to 1.5 times the IQR. Individual coloured lines represent individual participant data across conditions. Black diamonds denote the mean values for each condition. * Represents significance (*p* < 0.05).

Descriptive statistics of objective sleep outcomes, biometric measures, and daily perceived outcomes are displayed in Table 1. Differences in subjective daily perceived outcomes (calmness of sleep, ease of falling asleep, sleep satisfaction, and thermal comfort) are illustrated in Figure 1.

Linear mixed-model (LMM) results are shown in Table 2, Table 3, Table 4 and Table 5. The LMM identified significant main effects of condition on calmness of sleep (/5) (F (1, 252.29) = 8.01, *p* = 0.005), ease of falling asleep (/5) (F (1, 17.31) = 9.05, *p* = 0.008), ease of waking up (/5) (F (1, 18.95) = 6.49, *p* = 0.020), refreshed after waking (/5) (F (1, 20.07) = 14.33, *p* = 0.001) (Table 4), sleep satisfaction (/5) (F (1, 20.41) = 17.45, *p* < 0.001), thermal sensation (ASHRAE) (F (1, 22.19) = 10.84, *p* = 0.003), and thermal comfort (/5) (F (1, 20.15) = 41.53, *p* < 0.001) (Table 5). There were no other significant effects of condition on any objective sleep outcomes (TST, WASO, SOL, or SE) or biometric outcomes, including heart rate (HR), heart rate variability (HRV) or respiratory rate (RR) (Table 2 and Table 3).

Furthermore, the LMM demonstrated a significant order effect on participants’ ratings of their feelings of being refreshed after waking (/5) (F(1, 21.55 = 4.65, *p* = 0.001), whereby ratings were higher for participants who completed the POD condition first.

A significant effect of sex was reported for HRV (ms) (F (1, 22.60) = 5.45, *p* = 0.029), whereby HRV was higher in female participants compared to male participants.

Order-by-sex comparisons resulted in significant interaction effects for HRV (ms), with higher HRV in participants who completed the intervention first and were also female (F (1, 24.65) = 13.88, *p* = 0.001) (Table 3). Post hoc analysis revealed a trend between HRV in both females who completed the POD (mean difference = 34.015, SE = 7.982, *t* (23.023) = 4.261, *p* = 0.002) and CON (mean difference = 39.431, SE = 9.054, *t* (22.662) = 4.355, *p* = 0.001) condition first compared to males. Thermal sensation followed a similar trend, with ratings being higher in females who completed POD first (F (1, 25.51) = 4.44, *p* = 0.045) (Table 5). Post hoc analysis revealed no further significant trends.

Condition-by-order-by-sex comparisons revealed significant interaction effects for ratings of ease of falling asleep (F (1, 17.44) = 5.15, *p* = 0.036) (Table 4), with post hoc analysis reporting a significant trend for females in the CON condition who completed the CON condition first (mean difference = −1.202, SE = 0.348, *t* (38.757) = −3.449, *p =* 0.038). Further, an interaction effect was also found for thermal sensation (F (1, 22.21) = 10.02, *p* = 0.004) (Table 5). However, post hoc analysis revealed no further significant trends (*p ≥* 0.05). A significant interaction was reported for thermal comfort (F (1, 20.22) = 5.45, *p* = 0.030). Post hoc comparisons within the intervention-first order showed higher thermal comfort in the POD condition for males (mean difference = −1.596, SE = 0.327, *t*(20.752) = −4.882, *p* = 0.002).

Although not the main aim of the current study, the LMM also identified a significant effect of ambient temperature on HR and RR (Table 3), whereby higher ambient temperatures resulted in lower HR and higher RR.

### 2.3. Weekly Questionnaire

Descriptive statistics of weekly questionnaire measures are displayed in Table 6. Paired samples *t*-tests of self-reported sleep outcomes resulted in a significant difference in subjective sleep quality in favour of the POD condition, but not for any other perceptual measure.

### 2.4. Post-Data-Collection Questionnaire

In the post-data-collection questionnaire, 24 of 34 participants reported that they heard no noise from the temperature-regulated mattress cover when it was switched on. Of the remaining 10 participants who reported that they heard noise, 7 participants reported that the noise “did not impact their sleep”. In comparison, two participants reported that noise “slightly” impacted their sleep, and one participant reported that the noise “moderately” impacted their sleep. The average rating (whereby 1 = “impaired my sleep”, 5 = “no impact on my sleep”, and 10 = “improved my sleep”) of the mattress cover impacting participants’ sleep was 7.4 ± 1.5. The average rating (where 1 indicated that the participant “did not enjoy it and would not use it again” and 10 indicated that the participant “loved it and would definitely use it again”) of experience with the mattress cover was 7.9 ± 1.5.

## 3. Discussion

This study sought to determine the effect of a temperature-regulated mattress cover (POD) on sleep metrics, biometrics, and perceived sleep and comfort outcomes compared to a control (CON) condition. Overall, our results indicate that the temperature-regulated mattress cover was associated with significant benefits to the user’s daily perceived outcomes, including calmness of sleep, ease of falling asleep, ease of waking up, feeling refreshed after waking, sleep satisfaction, thermal sensation, and thermal comfort compared to the CON condition. However, these subjective improvements did not translate to objective sleep measures or biometric measures.

### 3.1. Perceived Outcomes

Previous studies using a temperature-controlled mattress cover from the same manufacturer as the current study reported greater odds ratios for calmer sleep (*p* = 0.04), ease of falling asleep (*p* = 0.02), and thermal comfort (*p* < 0.001) [18]. Similarly, the current study demonstrated significant benefits of the POD condition for these same outcomes. In addition to replicating these effects, the current study observed significant improvements in other perceived outcomes, including ease of waking up, feeling refreshed after waking, and sleep satisfaction, with *p*-values all < 0.05. While [18] found similar trends for these outcomes via odds ratios, their findings were not statistically significant but still support the notion that this technology may enhance perceptual outcomes. Participants in the current study completed sleep quality questionnaires at the end of each condition, and no significant differences were found in self-reported bedtime, SOL, waketime, or SE between conditions. However, this contrasts with previous researchers [18] who reported significantly shorter perceived SOL and improved objective SE when the temperature regulation feature was utilised. This discrepancy may reflect differences in study design or different sample populations. For instance, the current study followed a randomised, counterbalanced crossover design, whereas Moyen et al. [18] followed a repeated-measures, non-randomised design; followed a different condition order; and recruited broader populations (including perimenopausal and postmenopausal women).

The relationship between the bed microenvironment and sleep has been widely explored, particularly within the context of thermal comfort [9]. In the present study, subjective sleep outcomes improved in the POD condition despite minimal changes in objective sleep parameters. For instance, although participants reported finding it significantly easier to fall asleep in the POD condition, there were no significant differences in objective SOL measured via wrist actigraphy. Further, subjective ratings of overall sleep quality (from weekly questionnaires) were significantly higher in the POD condition, with *large* effect sizes, aligning with previous studies investigating thermoregulating bedding technologies on sleep quality [15,19]. This variance aligns with previous findings suggesting that subjective perceptions of sleep quality do not always correlate with objective measures [20]. Participants were aware of the condition they were undertaking (POD or CON), which may have amplified psychological factors such as expectancy of placebo effects. Given the absence of significant objective changes, it is plausible that improvements in sleep perception could be influenced by placebo mechanisms, consistent with previous sleep research reporting subjective benefits without corresponding objective outcomes [21]. Further, allowing participants to adjust their bed temperature in the POD condition may have enhanced perceived comfort through thermoregulatory mechanisms, even if these adjustments did not result in significant physiological changes. Prior studies have shown that thermal comfort and sensation positively influence sleep quality [22,23]. Additionally, despite reports of greater comfort and satisfaction, biometric indicators heart rate (HR), heart rate variability (HRV), and respiratory rate (RR) remained largely unchanged. This suggests the thermal environment may enhance perceived comfort without inducing significant physiological effects or altering sleep outcomes.

### 3.2. Objective Sleep Outcomes

The current study found no significant effects of the temperature-controlled mattress cover on objective sleep parameters, total sleep time (TST), sleep onset latency (SOL), or sleep efficiency (SE), a finding consistent with previous research using similar systems or high-heat-capacity mattress technology [13,17,18]. Furthermore, the disconnect between objective and subjective measures of sleep could be influenced by participants’ misperception of sleep quality. Studies have shown that sleep perception could affect psychosomatic responses. Rahman et al. [24] found that perceived sleep duration significantly influenced cognitive performance and waking EEG activity, with individuals performing better and showing altered brain activity patterns when they believed they had slept longer, regardless of actual sleep time [24]. In addition, the current study found no significant differences in wake after sleep onset (WASO), however, Moyen et al. [18] found that having the temperature setting activated resulted in significantly increased WASO by 8 min in women and an increased number of awakenings in men (+3 awakenings), although time spent in light and deep sleep increased. In contrast, studies investigating alternative temperature-regulating sleep technologies have reported significant improvements, including reductions in SOL and enhancements in overall measured sleep quality [15]. However, prolonged use of a temperature-controlled mattress cover may yield subtle improvements in sleep parameters that may become more pronounced over time. As such, longer studies are recommended to assess whether modest improvements develop into significant changes over time.

### 3.3. Biometric Outcomes

Exposure to high temperatures and high humidity has been shown to elevate heart rate, sweat rate, and thermal load, contributing to increased wakefulness [25]. In the current study, lower heart rates (HR) (−1 bpm, *p* = 0.051) were observed in the POD condition compared to CON, although the effect was *small*. While the reduction in HR was not significant in the current study, previous research [18] has reported a significant reduction in heart rate during sleep (−1.2 bpm, *p* < 0.001) when the temperature-regulation feature of the mattress cover was activated. Although other smart mattresses have demonstrated moderate to high validity in measuring sleeping HR, research on their test–retest reliability is limited, and small changes (e.g., 1 bpm) may fall within expected measurement variability rather than reflecting physiologically meaningful differences [26,27]. Previous studies have found lower sleeping HR when using high-heat-capacity mattresses to facilitate cooling [13,14]. These reductions in HR could be due to increased vagal activity, which activates warm-sensing and sleep-promoting neurons when exposed to variations in bed microenvironment temperature, thereby facilitating a more restorative sleep state for participants [4,13,14].

While heart rate variability (HRV) was not significantly affected in the present study, others [18] have observed significant changes in HRV (+2 ms in Pod) between Pod on versus Pod off conditions, with a mean difference of 7%. Authors from this study suggested that cardiovascular responses may be acutely sensitive to thermoregulatory bedding. Given our non-significant findings, further longitudinal studies are warranted to determine the sustained physiological impact of temperature-controlled mattress covers on autonomic regulation during sleep.

### 3.4. Ambient Temperature

Ambient temperature has been identified as a significant factor influencing subjective sleep quality, with prior studies reporting significant effects on calmness of sleep, ease of awakening, freshness after waking, and overall sleep satisfaction [22]. Although thermal comfort is often considered to be primarily driven by the bedding microenvironment, ambient conditions play an important role [28]. While the primary aim of the present study was not to assess the role of ambient temperature, the LMM revealed that ambient temperature significantly influenced biometric measures, with higher room temperatures linked to reduced HR and RR, but the effect was *small*. Few publications in the literature have addressed the effect of different ambient temperatures on cardiovascular responses during sleep, but one study is in contrast with these findings, reporting that lower ambient temperature (3 °C) significantly decreased HRV [29,30]. Previous literature has suggested that sleep is sensitive to nighttime ambient temperature and that environmental factors such as humidity, airflow, and temperature collectively affect sleep quality and comfort [4], but no sleep parameters were affected in the current study; therefore, further investigation into these effects is required. Notably, hot environments have been shown to disrupt sleep [4,31]. Our study included moderate ambient room temperatures; however, it would be interesting to evaluate the impact of this technology in warmer bedroom environments (e.g., >24 °C).

### 3.5. Limitations

This study employed a randomised, counterbalanced, crossover design and combined objective and subjective measures in a real-world setting, enhancing ecological validity. However, several limitations should be noted. The 7-day period per condition may have been insufficient to capture longer-term effects of the temperature-controlled mattress cover, particularly for objective outcomes. Additionally, objective measures of the bed (e.g., under the covers) microenvironment temperature were not recorded, limiting insight into the thermoregulatory mechanisms underlying the findings. Core and skin temperature measurement would also enhance understanding of the thermoregulatory mechanisms. Further, the menstrual cycle was not controlled for in the current study. Given that core body temperature varies across the menstrual cycle [32], future research should consider adjusting mattress cover temperature in accordance with the cycle phase to capture menstrual cycle-specific effects better. Data collection occurred in free-living conditions, and as such, questionnaire timing was not controlled. Although the average wake time was ~7 a.m., and the mean response time was 9:48 a.m., some participants may have responded immediately upon waking, when sleep inertia could have influenced their answers. Therefore, future research should control when daily questionnaires are completed by participants. Populations that may have challenges to their thermoregulation during sleep (e.g., elderly or individuals with spinal cord injuries) [30,33] may also benefit from this technology, warranting further research.

### 3.6. Main Conclusions

This study demonstrated that a temperature-controlled mattress cover was associated with improvements in all subjective sleep outcomes, thermal sensation, and comfort, and with weekly subjective sleep quality, though no significant changes were observed in objective sleep parameters or biometric indices. These findings are consistent with previous research suggesting that thermoregulatory interventions enhance perceived sleep quality, even when objective improvements were limited. Temperature-controlled mattress covers may serve as a non-invasive strategy to improve subjective sleep outcomes and thermal comfort, particularly for individuals experiencing thermal discomfort or challenges during sleep, those in thermally variable environments, athletes training or competing late at night (with increased core temperatures), the elderly, or those with disabilities that may impair thermoregulatory mechanisms (e.g., spinal cord injuries).

## 4. Materials and Methods

### 4.1. Participants

Sample size estimation was based on prior work investigating the effects of a temperature-controlled mattress cover on sleep outcomes in a crossover design [18]. In that study, a mean increase of 10 min in total sleep time (TST) was observed between conditions, and significant findings in subjective sleep data. Treating our randomised, counterbalanced crossover as a paired design using a linear mixed model (LMM) to account for within-subject repeated measures, a conservative estimate based on a two-tailed paired *t*-test indicated that 34 participants were required to detect this effect with 80% power at an alpha of 0.05 and an effect size of 0.50. This sample size is appropriate for detecting meaningful changes in sleep outcomes in a crossover design using LMMs.

A sample of 34 healthy, physically active adults (20 female, 14 male; mean ± age: 30.4 ± 5.7 y) participated in the current study. Participants were excluded from the study if they were unavailable for a 2–3-week participation period, were under 18 years of age, were pregnant, had an incompatible bed size, were partaking in shift work, took supplements/medications that affect sleep, and/or had diagnosed sleep disorders. Participants provided informed consent to participate in the study. The study was conducted in accordance with the Declaration of Helsinki, and ethical approval was obtained through the institution’s Human Research Ethics Committee (HEC24002). On average, participants partook in exercise 4 ± 2 days per week for 75 ± 51 min per day across the data collection period.

### 4.2. Study Design

**Figure 2:**
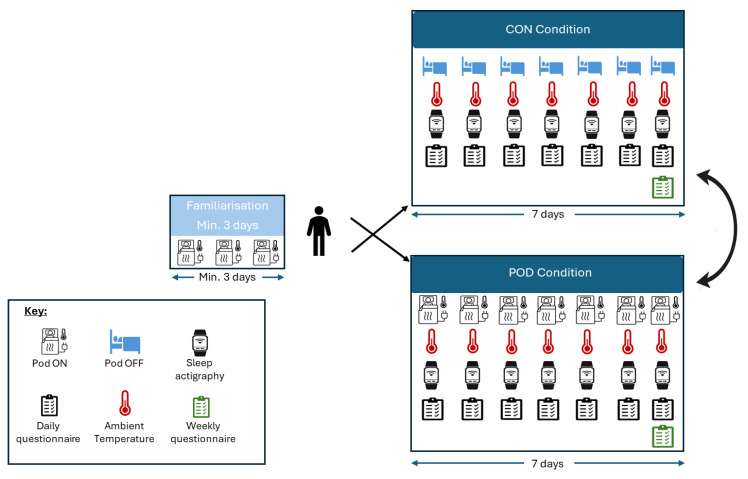
Schematic of the study protocol. This study followed a within-subject design. After a minimum of 3 nights of a familiarisation period sleeping on the mattress cover, participants (*n* = 34) were randomly assigned to spend 7 nights with the temperature regulation setting on (POD) and then another 7 nights with the temperature regulation setting off (CON) or vice versa (as indicated by far-right arrows). Sleep parameters, ambient temperature, relative humidity, and perceived outcomes were recorded throughout the 14-day period. A sleep quality questionnaire was completed at the end of each week.

The current study implemented a randomised, counterbalanced, crossover design (see Figure 2). Before data collection commenced, participants received a pre-testing introductory session where they received the equipment and were instructed on the use of all necessary devices (Eight Sleep Pod, Fitbit, and Govee digital weather meter). Participants were advised to continue with their normal habitual sleeping habits throughout the data collection period. Participants were encouraged to familiarise themselves with the temperature-controlled mattress cover and experiment with their own temperature preferences for different stages throughout the night. After a minimum of 3 nights of familiarisation, participants were instructed to sleep on the mattress cover for an additional 14 nights in their own home bedroom environment. Daily objective and subjective sleep measures, and measures of perceived comfort were assessed for 1 week with the temperature-regulation setting turned on (POD) and 1 week with the temperature-regulation setting turned off (CON) in a randomised, counterbalanced order. The room temperature and humidity of each participant’s sleeping environment were measured by a digital weather meter (H5179, Govee, Hong Kong, China). Participants were instructed to wear a wrist actigraphy device (Fitbit Charge 5, Fitbit San Francisco, CA, USA) every night throughout the study (14 nights). Participants completed weekly sleep quality questionnaires, including self-reported bed and wake times, sleep latencies, sleep duration, and ratings of sleep quality, as well as any adjustments to the temperature of the mattress cover twice throughout the data collection period at the end of week 1 and week 2. Lastly, participants recorded their exercise in the weekly questionnaire and were instructed to replicate it in the second week of data collection.

### 4.3. Temperature-Controlled Mattress Cover

**Figure 3:**
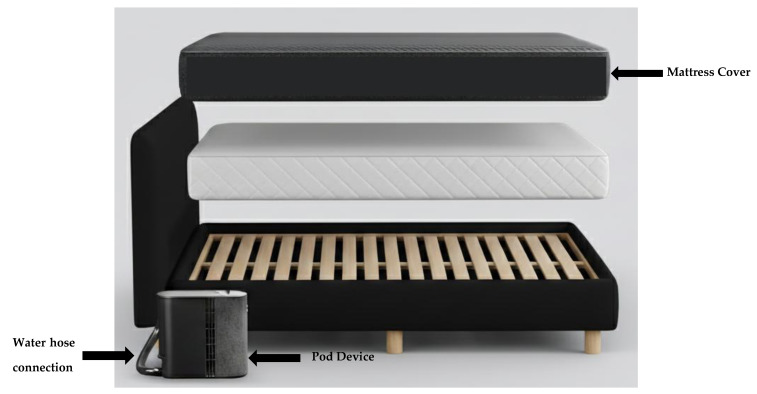
The temperature-controlled mattress cover and Pod device used in the current study. The system consists of a mattress cover connected via a water hose to the external Pod device, which regulates the bed temperature.

Participants were instructed to sleep on a temperature-controlled mattress cover or “Pod” (Eight Sleep, Pod 3, New York, NY, USA, Figure 3) to collect biometric data (heart rate, heart rate variability, respiratory rate, and sleep staging data). However, for the current study, sleep metrics were obtained using validated wrist actigraphy (see below). Heart rate (HR), heart rate variability (HRV), and respiratory rate (RR) were measured by the temperature-controlled mattress cover. According to the manufacturers, heart rate and heart rate variability have been validated against a gold-standard electrocardiogram (ECG), and the respiratory rate has been validated against the gold-standard respiratory inductance plethysmography belt, though this is yet to be validated externally. The temperature-controlled mattress cover controls temperature by continuously regulating water temperature flowing through the mattress cover to each individual’s preference while they sleep. The Pod consists of a hub (see Figure 3) that sits beside the bed and is connected to two embedded water tubes for active cooling and heating, which then connect to a cover that fits over the mattress like a thick fitted sheet. Inside the mattress cover, water flows through small tubes to cool the bed based on the selected setting (through a smartphone application).

Users can create a temperature profile on the smartphone application over three stages of the night, determined by the device’s software. The “Bedtime Phase” begins when the user goes to bed until after 15 min of consistent sleep is recorded. The “Early Phase” begins at the end of the Bedtime Phase, lasting 4 h, and lastly, the “Late Phase” begins at the end of the Early Phase and lasts until the user wakes. The water temperature circulating the mattress ranged from ~13 °C to 43 °C (or on a scale of −10 to +10, whereby −10 is indicative of the coldest setting, 0 is neutral, and +10 is the warmest setting), with the ability of each phase to change temperatures to the user’s preference. Even though Moyen et al. [18] recommended temperature settings of −1, 0, and +1 (26–29 °C) for women and −2, −1, and 0 (25–27 °C) temperature settings for men, participants were allowed to adjust their temperature profile via the smartphone application as desired throughout the POD condition. Once the temperature profile was set up for the participants completing the POD condition, the device automatically “primed” the bed approximately one hour before each participant’s specified bedtime to ensure it was at the set temperature before participants got into bed. In the CON condition, participants deactivated the temperature-control feature via the smartphone application to ensure that the temperature regulation did not occur. Researchers checked to make sure protocols for both POD and CON were adhered to by having the temperature feature on or off, respectively. There was a 100% adherence with the outlined protocols. The mattress cover also contains an AI-driven Autopilot feature designed to automatically adjust bed temperature during a sleep episode via multiple data streams (e.g., user sleep stage estimates, heart rate/heart rate variability, sleep/wake timing, room temperature and humidity, respiratory rate, and presence). During the familiarisation period, participants were instructed to adjust the temperature settings to achieve their preferred thermal comfort range. Once their preferred range was established, participants were less likely to make further manual adjustments during the data collection period, although changes remained possible if needed.

### 4.4. Sleep Monitoring

Participants were instructed to wear an actigraphy device (Fitbit Charge 5, Fitbit Inc., San Francisco, CA, USA) on their wrist to measure sleep. The Fitbit device was worn on either the dominant or non-dominant wrist, whichever was the most comfortable for the individual [34]. The Fitbit is equipped with a triaxial accelerometer and utilises Bluetooth to connect and transmit data to a smartphone through a dedicated software application. Previous research has shown that wrist-worn actigraphy devices from the same manufacturer exhibit no significant differences (*p* ≥ 0.05) in sleep onset latency and total sleep time compared to the gold-standard polysomnography (PSG) in healthy adults [35], and when comparing different brands of wearables against PSG, it was reported that Fitbit had a moderate accuracy in assessing total sleep time [36]. Further studies have indicated that devices from this manufacturer outperform various other wearable devices, including research-grade actigraphy devices, making them suitable for determining sleep duration in human participants [37]. In the current study, sleep outcomes, including total sleep time (TST), total time in bed (TTB), sleep efficiency (SE), sleep latency (SL), sleep time (ST), and wake time (WT), were utilised to evaluate participants’ sleep quality and quantity. These metrics were selected for their recognised accuracy in actigraphy-based assessments when compared to polysomnography [35].

### 4.5. Sleep Regularity Index (SRI)

The Sleep Regularity Index (SRI) measures how consistent an individual’s sleep and wake times are on a night-to-night basis, based on variations in sleep onset and wake times [38]. It represents the probability that an individual is in the same state (asleep or awake) at any given time on successive days. This probability is calculated across the full duration of the assessment period to yield a single SRI score. An SRI value of 100 is indicative of an identical sleep–wake cycle between consecutive days, whereas a score of 0 reflects complete irregularity with no overlap in sleep–wake states across days, with higher SRI scores reported to relate to better sleep outcomes [39]. SRI values for each participant were calculated using binary sleep–wake data. Sleep onset and wake times were identified from wrist actigraphy and converted to UNIX timestamps, representing both date and time. Each timestamp was then coded as “1” for sleep and “0” for wake. SRI values were calculated with the R (RStudio) statistical software (version 2025.05; R Foundation for Statistical Computing, Vienna, Austria), using the *sleepreg* package [40].

### 4.6. Bedroom Environment

Temperature and environmental data were recorded using a Govee digital weather meter (H5179, Govee, Hong Kong, China), which measures air temperature and humidity. Participants were instructed to place the digital weather meter as close to their bed as possible to measure the ambient room temperature of the participant’s sleep environment. According to the manufacturers, the digital weather meter has a temperature range of −20 °C to 60 °C and an accuracy of ±0.3 °C for temperature and ±3% relative humidity (RH) between these temperatures. The Govee device utilises Bluetooth and Wi-Fi to connect and transmit data to a smartphone device via a dedicated software application. While validation studies are not yet available, these devices have been utilised in previous research to measure thermal comfort in classroom settings [41].

### 4.7. Menstrual Cycle Questionnaire

Female participants completed a menstrual cycle questionnaire before data collection via REDCap (Research Electronic Data Capture, Vanderbilt, TN, USA). Questionnaires collected the date of last menstruation, which was then used to categorise each day of data collection into menstrual phases, in accordance with Najmabadi et al. [42]. Female participants were also questioned on their menstrual cycle length, the presence of any menstrual disorders, and the use of hormonal contraceptives.

### 4.8. Perceived Outcomes

#### 4.8.1. Daily Perceived Outcomes

A daily questionnaire was administered to participants every morning during data collection via REDCap (Research Electronic Data Capture, Vanderbilt, TN, USA) at ~6:30 a.m., with an average response time of 9:48 a.m., suggesting that participants likely filled out the questionnaire after the dissipation of sleep inertia. The questionnaire included a series of questions regarding each participant’s sleep and thermal comfort related to their sleep the night before. Questions using a 5-point Likert scale were related to the following topics: calmness of sleep (1 = very restless, 2 = somewhat restless, 3 = neutral, 4 = somewhat calm, and 5 = very calm), ease of falling asleep and waking up (1 = very difficult, 2 = difficult, 3 = neutral, 4 = easy, and 5 = very easy), how refreshed they felt after waking (1 = not refreshed at all, 2 = slightly refreshed, 3 = moderately refreshed, 4 = very refreshed, and 5 = extremely refreshed), and sleep satisfaction (1 = very dissatisfied, 2 = dissatisfied, 3 = neutral, 4 = satisfied, and 5 = extremely satisfied). Furthermore, thermal sensation was measured using the ASHRAE 7-point thermal sensation scale [43] with the following weighting for each response (−3 = cold, −2 = cool, −1 = slightly cool, 0 = neutral, 1 = slightly warm, 2 = warm, and 3 = hot) and with the user’s ratings of comfort with their thermal sensation using a 5-point Likert scale (1 = very uncomfortable, 2 = uncomfortable, 3 = neutral, 4 = comfortable and 5 = very comfortable). Participants were also asked to provide any further comments on their sleep episode.

#### 4.8.2. Weekly Questionnaire

Participants filled out a weekly questionnaire at the end of each condition week (POD and CON). This questionnaire was a variation of the Brief Pittsburgh Sleep Quality Index (B-PSQI) [44] with the wording amended for participants to reflect on their sleep over the last week rather than the last month. The sleep quality questionnaire contained six items enquiring about bedtime, wake time, sleep latency, sleep duration, wake after sleep onset, and sleep quality. Similar to Moyen et al. [18], sleep disturbances were not included, as the validated scoring required responses reflecting “over one month” rather than the assessment “over the past week” in the current study. Lastly, the weekly questionnaire included items assessing whether participants adjusted the temperature settings of the Pod during the POD condition.

#### 4.8.3. Post-Data-Collection Questionnaire

After the second week of data collection, the weekly questionnaire was administered again to each participant with additional questions on each participant’s experience with the temperature-controlled mattress cover. Participants were required to self-report if they heard noise from the Pod when completing the POD condition. If yes, they were prompted to review the noise of the POD affecting their sleep on a 5-point Likert scale whereby 1 = “not at all” and 5 = “extremely”. Questions enquired if they thought the mattress cover impacted their sleep on a scale of 1 to 10—whereby 1 = “impaired my sleep”, 5 = “no impact on my sleep”, and 10 = “improved my sleep”—and also participant’s satisfaction with the use of the mattress cover on a scale of 1 to 10 where 1 = “did not enjoy it and would not use it again” and 10 = “loved it and would definitely use it again”. Questionnaire data were captured using REDCap (Research Electronic Data Capture, Vanderbilt, TN, USA).

### 4.9. Statistical Analysis

Data analysis was conducted on Jamovi (Jamovi Version 2.3.28, Jamovi Project 2018), with statistical significance set at *p* < 0.05. The results are presented as mean ± standard deviations (SD). Before analysis, data were checked for outliers by visual inspection of a box plot and quantile–quantile plot, and data normality was checked via Shapiro–Wilk tests. Outlier detection was carried out via the interquartile range approach, whereby the interquartile range was calculated for each dependent variable measured. Any datapoints outside of the interquartile range were considered as outliers. A total of 26 nights were removed across the entire 476 nights of data collection due to outliers or missing data.

Linear mixed-model (LMM) analyses were used to evaluate differences in objective and daily subjective sleep parameters and biometric measures in the POD and CON conditions. The analysis included fixed factors: condition (POD or CON), order (POD first vs. CON first) and sex (male–female), with covariates, including ambient temperature and relative humidity. Participants were included as a random effect for all LMM analyses. Statistical significance for fixed effects was determined using F tests with the degrees of freedom for F statistics computed using the Satterthwaite approximation method. Assumptions of normality and homoscedasticity were assessed using model residuals. Models that failed to converge were iteratively re-fitted using alternative optimisers. Paired samples *t*-tests with significance set at *p* < 0.05 were conducted on weekly sleep quality questionnaire data, with Bonferroni–Holm correction applied to adjust for multiple comparisons and reduce the risk of type 1 error. Effect sizes were calculated using Cohen’s *d* with the standard errors provided for weekly questionnaire data. Standardised mean difference (SMD) and effect size thresholds were based on Cohen’s *d* and set at <0.2—*trivial*, 0.2—*small*, 0.5—*moderate*, and 0.8—*large* [45]. Where 95% confidence limits overlapped the thresholds for *small* positive and *small* negative values, the effect was considered *unclear*.

## Figures and Tables

**Table 1:** Descriptive statistics, including the mean and standard deviation of all daily measures (among objective sleep outcomes, biometric measures, and daily perceived outcomes) in both the CON and POD conditions.

Variable	CON Mean ± SD	POD Mean ± SD
**Objective sleep outcomes**		
Total sleep time (h:mm)	7 h 25 min ± 32 min	7 h 34 min ± 33 min
Wake after sleep onset (h:mm)	1 h 1 min ± 13 min	1 h 4 min ± 13 min
Sleep onset latency (min)	11 ± 8	10 ± 6
Sleep efficiency (%)	87 ± 2	87 ± 2
Sleep regularity index (/100)	74.1 ± 10.7	71.9 ± 9.4
**Biometric outcomes**		
Heart rate (bpm)	54 ± 6	53 ± 6
Heart rate variability (ms)	66 ± 25	68 ± 25
Respiratory rate (breaths per min)	15 ± 2	15 ± 2
**Daily perceived outcomes**		
Calmness of sleep (/5)	3.4 ± 0.7	3.7 ± 0.7 *
Ease of falling asleep (/5)	3.6 ± 0.6	3.9 ± 0.6 *
Ease of waking up (/5)	3.0 ± 0.6	3.2 ± 0.6 *
Refreshed after waking (/5)	2.7 ± 0.6	3.1 ± 0.7 *
Sleep satisfaction (/5)	3.3 ± 0.5	3.7 ± 0.5 *
Thermal sensation (ASHRAE)	0.5 ± 0.8	−0.1 ± 1.0 *
Thermal comfort (/5)	3.3 ± 0.6	4.0 ± 0.5 *

* Indicates significant difference between conditions (*p* < 0.05).

**Table 2:** Linear mixed-model interaction, including the estimate, *p*-value, and standardised mean difference (SMD) for the following objective outcomes: total sleep time (TST), wake after sleep onset (WASO), sleep onset latency (SOL), and sleep efficiency (SE). Bold text indicates significant findings (*p* < 0.05).

Predictor	Estimate (B) (95% CI)	*p*-Value	SMD	Estimate (B) (95% CI)	*p*-Value	SMD	Estimate (B) (95% CI)	*p*-Value	SMD	Estimate (B) (95% CI)	*p*-Value	SMD
	**TST (min)**			**WASO (min)**			**SOL (min)**			**SE (%)**		
Intercept	455.364 (441.209–469.519)			65.487 (59.128–71.847)			10.706 (7.613–13.799)			87.602 (86.736–88.470)		
Condition	12.082 (−1.207–25.370)	0.094	0.229	5.110 (0.193–10.026)	0.054	0.360	−1.303 (−3.752–1.147)	0.300	−0.161	−0.557 (−1.470–0.356)	0.244	−0.225
Order	−0.235 (−28.442–27.971)	0.987	−0.005	3.963 (−8.617–16.544)	0.542	0.280	−0.607 (−6.784–5.571)	0.848	−0.075	−0.822 (−2.530–0.887)	0.355	−0.335
Ambient Temperature	1.332 (−3.094–5.759)	0.554	0.026	1.303 (−0.167–2.773)	0.082	0.269	0.696 (−0.071–1.463)	0.076	0.249	−0.126 (−0.365–0.113)	0.300	−0.154
Relative Humidity	0.414 (−0.950–1.777)	0.551	0.008	−0.103 (−0.559–0.353)	0.656	−0.060	−0.156 (−0.395–0.083)	0.201	−0.157	0.025 (−0.052–0.101)	0.527	0.083
Sex	1.508 (−26.795–29.811)	0.917	0.029	6.104 (−6.507–18.715)	0.352	0.431	0.480 (−5.710–6.669)	0.880	0.059	−0.950 (−2.663–0.763)	0.288	−0.389
Condition × Order	16.784 (−10.369–43.937)	0.241	0.319	−8.920 (−18.923–1.083)	0.093	−0.629	−2.884 (−7.873–2.106)	0.260	−0.353	1.958 (0.107–3.809)	0.050	0.797
Condition × Sex	27.351 (0.622–54.079)	0.062	0.518	0.842 (−9.034–10.717)	0.868	0.059	−1.809 (−6.715–3.096)	0.471	−0.223	0.763 (−1.070–2.596)	0.422	0.311
Order × Sex	28.593 (−31.722–88.908)	0.359	0.554	9.081 (−16.745–34.906)	0.496	0.642	2.848 (−10.054–15.751)	0.667	0.348	−1.327 (−4.870–2.215)	0.469	−0.540
Condition × Order × Sex	−19.674 (−72.903–33.555)	0.478	−0.373	8.820 (−10.869–28.508)	0.388	0.623	0.224 (−9.596–10.043)	0.964	0.029	−2.388 (−6.044–1.267)	0.213	−0.978
**Random Effects**												
Between subjects SD	31.453			14.277			7.244			1.870		
Within subjects SD	9.943			7.198			3.010			1.481		
ICC	0.283			0.523			0.458			0.388		

**Table 3:** Linear mixed model interaction, including the estimate, *p*-value, and standardised mean difference (SMD) for the following biometric outcomes: heart rate (HR), heart rate variability (HRV), and respiratory rate (RR). Bold text indicates significant findings (*p* < 0.05).

Predictor	Estimate (B) (95% CI)	*p*-Value	SMD	Estimate (B) (95% CI)	*p*-Value	SMD	Estimate (B) (95% CI)	*p*-Value	SMD
	**HR (bpm)**			**HRV (ms)**			**RR (/min)**		
Intercept	54.556 (52.502–56.610)			63.706 (57.422–69.990)			15.268 (14.367–16.170)		
Condition	−1.241 (−2.422–−0.060)	0.051	−0.404	3.457 (−0.572–7.4986)	0.107	0.272	−0.100 (−0.276–0.075)	0.273	−0.197
Order	0.474 (−3.632–4.579)	0.822	0.153	−9.505 (−22.067–3.056)	0.150	−0.749	−1.410 (−3.214–0.393)	0.138	−2.779
Ambient Temperature	−0.373 (−0.699–−0.047)	**0.025**	−0.354	0.354 (−0.938–1.647)	0.590	0.081	−0.066 (−0.122–−0.010)	**0.021**	−0.375
Relative Humidity	−0.034 (−0.131–0.063)	0.492	−0.092	−0.075 (−0.456–0.306)	0.699	−0.048	0.005 (−0.021–0.012)	0.556	−0.079
Sex	0.085 (−4.026–4.196)	0.968	0.026	−14.922 (−27.509–−2.335)	**0.029**	−1.176	−0.683 (−2.487–1.120)	0.464	−1.340
Condition × Order	−1.077 (−3.471–1.317)	0.385	−0.348	−2.195 (−10.403–6.013)	0.604	−0.173	−0.361 (−0.718–−0.003)	0.059	−0.710
Condition × Sex	−0.857 (−3.230–1.515)	0.485	−0.280	1.624 (−6.482–9.730)	0.697	0.128	−0.300 (−0.653–0.052)	0.109	−0.591
Order × Sex	−7.597 (−15.963–0.770)	0.087	−2.472	49.018 (23.115–74.922)	**0.001**	3.864	0.019 (−3.599–3.637)	0.992	0.039
Condition × Order × Sex	0.664 (−4.064–5.392)	0.785	0.221	−2.654 (−18.786–13.478)	0.749	−0.209	−0.023 (−0.726–0.680)	0.949	−0.039
**Random Effects**									
Between subjects SD	5.062			15.252			2.266		
Within subjects SD	2.147			5.999			0.291		
ICC	0.744			0.607			0.955		

**Table 4:** Linear mixed-model interaction, including the estimate, *p*-value, and standardised mean difference (SMD) for the following subjective perceived outcomes: calmness of sleep, ease of falling asleep, ease of waking up, and being refreshed after waking. Bold text indicates significant findings (*p* < 0.05).

Predictor	Estimate (B) (95% CI)	*p*-Value	SMD	Estimate (B) (95% CI)	*p*-Value	SMD	Estimate (B) (95% CI)	*p*-Value	SMD	Estimate (B) (95% CI)	*p*-Value	SMD
	**Calmness of Sleep (/5)**			**Ease of Falling Asleep (/5)**			**Ease of Waking Up (/5)**			**Refreshed After Waking (/5)**		
Intercept	3.710 (3.447–3.973)			3.843 (3.658–4.027)			3.226 (3.005–3.447)			3.029 (2.770–3.289)		
Condition	0.403 (0.123–0.684)	**0.005**	0.347	0.370 (−0.128–0.613)	**0.008**	0.384	0.363 (0.082–0.643)	**0.020**	0.417	0.500 (0.240–0.759)	**0.001**	0.607
Order	0.373 (−0.150–0.897)	0.174	0.329	0.263 (−0.104–0.630)	0.172	0.271	0.386 (−0.055–0.826)	0.100	0.440	0.566 (0.049–1.083)	**0.042**	0.681
Ambient Temperature	−0.027 (−0.120–0.065)	0.564	−0.070	−0.010 (−0.083–0.062)	0.786	−0.021	0.020 (−0.054–0.093)	0.597	0.059	−0.034 (−0.107–0.040)	0.368	−0.123
Relative Humidity	−0.004 (−0.032–0.024)	0.776	−0.026	−0.006 (−0.028–0.016)	0.578	−0.053	−0.007 (−0.029–0.016)	0.569	−0.059	−0.005 (−0.027–0.018)	0.681	−0.049
Sex	0.159 (−0.366–0.684)	0.557	0.139	0.289 (−0.079–0.657)	0.136	0.302	0.188 (−0.253–0.630)	0.411	0.220	0.247 (−0.271–0.766)	0.358	0.292
Condition × Order	−0.217 (−0.789–0.356)	0.457	−0.191	0.014 (−0.479–0.507)	0.956	0.010	0.215 (−0.352−0.782)	0.464	0.244	0.120 (−0.407–0.646)	0.659	−0.146
Condition × Sex	0.259 (−0.305–0.824)	0.367	0.226	0.476 (−0.012–0.963)	0.071	0.499	0.137 (−0.426–0.700)	0.637	0.162	0.009 (−0.512–0.531)	0.972	0.024
Order × Sex	0.697 (−0.534–1.729)	0.308	0.521	−0.141 (−0.944–0.663)	0.733	−0.146	−0.277 (−1.221–0.667)	0.569	−0.325	0.727 (−0.364–1.817)	0.202	0.888
Condition × Order × Sex	0.805 (−0.320–1.929)	0.160	0.704	1.119 (0.148–2.090)	**0.036**	1.176	−0.097 (−1.219–1.026)	0.867	−0.093	0.874 (−0.1767–1.914)	0.114	1.058
**Random Effects**												
Between subjects SD	0.550			0.349			0.476			0.592		
Within subjects SD	0.030			0.160			0.439			0.387		
ICC	0.196			0.124			0.246			0.356		

**Table 5:** Linear mixed-model interaction, including the estimate, *p*-value, and standardised mean difference (SMD) for the following subjective perceived outcomes: sleep satisfaction scored on a 5-point Likert scale, thermal sensation scored on a 7-point Likert scale, and thermal comfort scored subjectively on 5-point Likert scale. Bold text indicates significant findings (*p* < 0.05).

Predictor	Estimate (B) (95% CI)	*p*-Value	SMD	Estimate (B) (95% CI)	*p*-Value	SMD	Estimate (B) (95% CI)	*p*-Value	SMD
	**Sleep Satisfaction (/5)**		**Thermal Sensation (/7)**			**Thermal Comfort (/5)**			
Intercept	3.618 (3.429–3.806)			0.139 (−0.108–0.387)			3.708 (3.535–3.881)		
Condition	0.464 (0.245–0.683)	**<0.001**	0.523	−0.739 (−1.181–−0.297)	**0.003**	−0.926	0.912 (0.633–1.191)	**<0.001**	1.194
Order	0.313 (−0.062–0.687)	0.115	0.352	0.104 (−0.389–0.598)	0.682	0.140	0.306 (−0.039–0.650)	0.096	0.407
Ambient Temperature	−0.044 (−0.113–0.025)	0.215	−0.148	0.042 (−0.029–0.114)	0.247	0.152	0.029 (−0.034–0.091)	0.366	0.105
Relative Humidity	0.001 (−0.022–0.020)	0.921	−0.011	−0.000 (−0.023–0.023)	0.999	0.000	0.005 (−0.015–0.024)	0.635	0.052
Sex	0.177 (−0.199–0.553)	0.363	0.204	−0.186 (−0.682–0.309)	0.466	−0.241	0.015 (−0.330–0.361)	0.931	0.013
Condition × Order	−0.215 (−0.661–0.230)	0.352	−0.238	−0.236 (−1.123–0.651)	0.605	−0.304	0.291 (−0.271–0.853)	0.320	0.380
Condition × Sex	0.213 (−0.226–0.653)	0.350	0.238	−0.443 (−1.329–0.442)	0.335	−0.558	0.416 (−0.143–0.975)	0.158	0.551
Order × Sex	0.422 (−0.392–1.236)	0.317	0.476	−1.115 (−2.158–−0.073)	**0.045**	−1.409	−0.253 (−0.998–0.492)	0.510	−0.354
Condition × Order × Sex	0.815 (−0.061–1.691)	0.081	0.919	−2.842 (−4.609–−1.074)	**0.004**	−3.602	1.322 (0.207–2.438)	0.030	1.719
**Random Effects**									
Between subjects SD	0.382			0.553			0.355		
Within subjects SD	0.112			0.962			0.503		
ICC	0.169			0.344			0.190		

**Table 6:** Mean and standard deviation of self-reported sleep outcomes from the weekly questionnaire in both the CON and POD conditions, along with p-values from paired samples *t*-tests and Cohen’s *d* effect sizes (with 95% confidence intervals). Bold text indicates significant findings (*p* < 0.05).

Variable	CON Mean ± SD	POD Mean ± SD	*p*-Value	Effect Size (95% CI)
Self-reported bedtime (hh:mm)	22:31 ± 0:46	22:32 ± 0:45	0.70	0.00 (±0.33) *unclear*
Self-reported waketime (hh:mm)	07:05 ± 0:54	07:02 ± 0:55	0.78	−0.05 (±0.20) *trivial*
Self-reported sleep onset latency (min)	22 ± 11	20 ± 9	0.37	−0.13 (±0.23) *trivial*
Self-reported total sleep time (h:min)	7 h 17 min ± 32 min	7 h 28 min ± 35 min	0.52	0.27 (±0.17) *small*
Self-reported sleep quality (/5)	2.8 ± 0.5	3.2 ± 0.5	**<0.001**	0.92 (±0.40) *large*

## Data Availability

The original contributions presented in this study are included in the article. Further inquiries can be directed to the corresponding authors.
